# Pulmonary Complications in Fatal Yellow Fever, Brazil, 2017–2019

**DOI:** 10.3201/eid3202.250530

**Published:** 2026-02

**Authors:** Amaro N. Duarte-Neto, Katia C. Dantas, Suzette C. Ferreira, Fernando R. Giugni, Marielton P. Cunha, Shahab Z. Pour, Felipe L. Ledesma, Yeh-Li Ho, Ana C.S. Nastri, Cinthya S.C. Borges, Fernanda A. Rodrigues, Ceila M.S. Malaque, Jaques Sztajnbok, Thais Mauad, Luiz F.F. Silva, Paulo H.N. Saldiva, Marisa Dolhnikoff

**Affiliations:** Universidade de São Paulo, São Paulo, São Paulo, Brazil (A.N. Duarte-Neto, K.C. Dantas, F.R. Giugni, M.P. Cunha, S.Z. Pour, F.L. Ledesma, Y.-L. Ho, A.C.S. Nastri, F.A. Rodrigues, T. Mauad, L.F.F. Silva, P.H.N. Saldiva, M. Dolhnikoff); Fundação Pró-Sangue Hemocentro de São Paulo, São Paulo (S.C. Ferreira); Instituto Adolfo Lutz, São Paulo (C.S.C. Borges); Instituto de Infectologia Emílio Ribas, São Paulo (C.M.S. Malaque, J. Sztajnbok).

**Keywords:** yellow fever, viruses, pneumonia, yellow fever vaccine, autopsy, fungi, bacteria, fungal infections, vector-borne infections, sepsis, epidemic, Brazil

## Abstract

Yellow fever (YF) mainly causes severe hepatitis; data on pulmonary pathology remain limited. We investigated respiratory tract pathology in 73 fatal YF cases during the 2017–2019 epidemic in São Paulo, Brazil. All patients died from YF-related fulminant hepatitis. Autopsies revealed frequent tracheitis (91%), pulmonary edema and hemorrhage (100%), diffuse alveolar damage (84%), secondary pneumonia (84%), and bronchoaspiration (60%). Microabscesses, thrombi, and hemophagocytosis were also observed. In 5 cases of vaccine-associated viscerotropic disease, hemorrhage and diffuse alveolar damage were prominent. We detected antigens of YF virus in all cases and viral RNA in 94%. Molecular analysis identified bacterial and fungal pathogens in pneumonia, including gram-negative bacilli, *Candida* spp., and *Aspergillus* spp. Electron microscopy did not reveal viral particles in 3 examined cases. Our findings underscore the respiratory tract involvement in severe YF and could help guide diagnosis, intensive care, and public health preparedness in future outbreaks.

Yellow fever (YF) is a mosquitoborne, flavivirus-induced hemorrhagic fever with a high case-fatality rate. The disease is endemic in tropical regions of South America and sub-Saharan Africa, with sporadic epidemics (*1*,*2*).

Since the end of 2016, YF has reemerged in Brazil, affecting areas not previously considered to be at risk outside the Amazon Basin, the region in Brazil endemic for the disease (*3*). During 2017–2019, a total of 696 cases of YF were reported in the state of São Paulo, with 232 deaths (mortality rate 33.3%) (*4*–*6*). Several factors are likely to have contributed to the recent expansion of YF in Brazil toward the southeast region. In São Paulo state, the vector, *Aedes aegypti* mosquitoes, has reached near-universal distribution, infesting ≈93.6% of municipalities. That pattern is exacerbated during warmer and wetter periods, such as El Niño events, and is strongly associated with urban density and inadequate infrastructure. Other contributing factors include the increase in neotropical nonhuman primates as susceptible amplifier hosts of YF virus (YFV), low vaccination coverage of susceptible persons living close to the forest, and viral factors. Molecular sequencing and phylogenetic studies have demonstrated that the South American genotype I, lineage 1E, is the strain of YFV involved in recent Brazil epidemics; some mutations are potentially influencing viral replication and fitness (*3*–*5*).

The clinical picture of YF is classified in 5 periods: the incubation period, after the bite of an infected mosquito; the period of infection, which coincides with viremia, with influenza-like symptoms; the period of remission, in which the patient has a slight improvement in symptoms and might progress to cure, and in which serum YFV IgM neutralizing antibodies appear; the period of intoxication, with variable severity, characterized by recurrence of fever, signs and symptoms of an acute hepatitis, oliguria, and hemorrhagic phenomena; and finally, the period of convalescence, with complete resolution of symptoms and laboratory alterations (*2*,*7*).

The pathology of severe YF, first described in the 19th Century, consists of midzonal hepatitis as the main lesion; it is characterized by apoptotic and steatotic hepatocytes associated with visceral edema and hemorrhagic phenomena, which lead to death (*8*,*9*). However, studies conducted during the 2017–2019 YF epidemic in Brazil have broadened our understanding of the disease (*7*,*10*–*16*). Those investigations have provided new insights into the molecular characteristics of circulating YFV strains; emerging clinical manifestations including seizures, pancreatitis, refractory acidosis, and late-onset hepatitis recurrence in convalescent patients; the kinetics of serum YFV RNA detected by reverse transcription PCR (RT-PCR); mortality predictors and prognostic biomarkers; and novel therapeutic strategies such as hemodialysis and plasma exchange. In addition, new data on the pathology of YF have been described. Our group has previously demonstrated YFV replication in various body systems (*5*), characterization of YFV-infected liver grafts in cases undergoing liver transplantation for YFV fulminant hepatitis (*17*), ultrasound-guided minimally invasive tissue sampling as an equivalent alternative to conventional autopsy for the postmortem diagnosis of YF (*9*), and a detailed description of cardiac and endothelial pathology in YF (*18*).

Although YFV is considered a hepatotropic virus, the systemic effects of severe YF are not yet well understood. One of the most intriguing findings from our autopsy cohort of fatal YF cases is pulmonary injury. In previous studies describing the first 20 cases, we reported severe pneumonia caused by bacteria and filamentous fungi in 12 cases, in addition to alveolar edema and hemorrhage in all 20 (*9*,*17*). To understand the role of the respiratory tract in the pathology of severe or fatal YF, we described the tracheal and pulmonary pathological changes in 73 fatal cases, their association with clinical and laboratory features, and molecular findings.

## Methods

### Study and Autopsy Protocols

This autopsy-based case series study was conducted during the seasonal period of 2017–2019 YF epidemic. This report is part of a project approved by Hospital das Clínicas da Faculdade de Medicina da Universidade de São Paulo (HCFMUSP) Ethical Committee (process no. 2·669·963). That hospital and Emílio Ribas Institute of Infectious Diseases are referral hospitals for YF cases in the state of São Paulo, Brazil; all YF cases were referred to them. The committee recommended autopsy upon patient death; all autopsies were performed with the written consent of first-degree relatives.

We used the definition from the Brazil Ministry of Health and local public health authorities to diagnose YF (*6*,*7*,*10*). Confirmed cases had a compatible clinical manifestation and laboratory confirmation by >1 method: positive serum IgM, detection of YFV RNA by RT-PCR in blood samples, and histopathology compatible for YF hepatitis with YF antigen detectable in tissues by immunohistochemistry (*6*,*7*,*10*,*11*). We excluded other causes of hepatic failure (Appendix). We classified the YF vaccine–associated viscerotropic disease (YEL-AVD) as level 1 of diagnostic certainty (*19*). 

We collected demographic and clinical data from patients’ records; data included sex (assigned at birth), age, previous medical history, and clinical and laboratory results during hospitalization. We performed autopsies in accordance with Letulle’s technique and examined all organs (*9*). From each patient we collected >10 lung samples, including 1 peripheric (with pleural tissue) and 1 from the central area of each pulmonary lobe. In 57 (78%) cases, we took trachea samples. We fixed tissue samples in buffered 10% formalin, embedded them in paraffin sections, and used hematoxylin and eosin (HE) stain. We performed additional stains in pulmonary sections with histologic signs of infections. A pathologist specializing in autopsy and infectious diseases and several pulmonary pathologists reviewed the histopathology of all samples; we resolved disagreements by consensus.

### Molecular Diagnosis and Sequencing

We collected a total of 73 lung tissue samples during autopsy and used specific primers and probes in quantitative RT-PCR to detect YFV RNA (*5*,*9*,*17*). We performed nested PCR in frozen lung samples for fungi and bacteria detection. We Sanger sequenced samples of lung tissue in which fungi and bacteria were detected (Appendix).

### Immunohistochemistry

Selected pulmonary cuts without extensive pneumonia underwent immunohistochemistry reactions for detecting YFV antigens. We used a primary antibody, a polyclonal anti-YFV mouse ascitic fluid specific to the virus, originally standardized for ELISA and validated for formalin-fixed paraffin-embedded tissues in our laboratories at the optimized dilution of 1:20,000 (*9*,*17*). In the validation procedures, we tested YFV primary antibody on YFV-infected liver samples confirmed by PCR and serology and in negative cases with different liver diseases for specificity (Appendix). We considered samples positive for YFV if chromogen stained the cytoplasm of hepatocytes, Kupffer cells, or inflammatory cells in the liver. All YF-negative cases had a negative reaction for YFV antigens in the liver. We also tested for vascular cell adhesion molecule (VCAM, mouse monoclonal, clone E10; Santa Cruz Biotechnology, Inc., https://www.scbt.com) at 1:100 dilution and VIII factor of coagulation (polyclonal IH, 760–2642; Roche, https://www.roche.com). We have not performed immunohistochemistry reactions to detect YFV antigens on tracheal sections.

### Electron Microscopy

We collected 3 lung tissue samples from different cases and fixed them in 3% glutaraldehyde for examination. We fixed and processed samples as previously described (*17*). We analyzed the thin sections under a transmission electron microscope (Philips Tecnai 10, 80kV; Thermo Fisher Scientific, https://www.thermofisher.com).

## Results

During 2017–2019, a total of 696 cases of YF were reported in the state of São Paulo; 232 (33.3%) cases resulted in death. Of those, 84 cases were referred for autopsy at the central morgue in São Paulo to determine cause of death; YF was excluded as the cause in 9 cases and confirmed in 73 cases. The main cause of death was fulminant YF-related hepatitis in all 73 cases. The immediate causes of death were as follows: pulmonary hemorrhage in 28 cases (38.4%), intraabdominal hemorrhage in 26 (35.6%), sepsis due to intestinal ischemia in 6 (8.2%), cerebral event (hemorrhage or herniation) in 6 (8.2%), necrohemorrhagic pancreatitis in 4 (5.5%), and sepsis due to bronchopneumonia in 3 cases (4.1%). Therefore, for 31 (42.4%) cases, the immediate cause of death was pneumonia or hemorrhage in the respiratory tract. The time from death to autopsy was 6 hours to 44 hours 19 minutes (median 14 hours 50 minutes).

### Clinical Characteristics and Laboratory Results

Of the 73 confirmed cases of YF, we classified 68 (93.2%) cases as wild-type YF, and 5 (6.8%) cases as YEL-AVD (Table 1). Sixty-two (84.9%) of the 73 patients were male and 11 (15.1%) female; median age was 48 (34–60) years. The most common underlying conditions were arterial hypertension (28.8%), diabetes (11%), alcohol use (50.7%), and smoking (37.0%). The median duration of hospitalization was 5 days, and the median interval from symptoms to death was 9 days. Four patients underwent liver transplantation for YF-fulminant hepatitis. Secondary infection was diagnosed clinically in 56 (76.7%) cases, mostly through blood cultures. All patients underwent mechanical ventilation and experienced shock, requiring vasopressors. Initial laboratory results showed neutrophilia (median 4,466 cells/µL, reference range 1,592-4,350 cells/µL), lymphopenia (median 675 cells/µL, reference range 1,120–2,946 cells/µL), and ratio of the partial pressure of oxygen in arterial blood to inspired oxygen (PaO_2_/FiO_2_) >300 mm Hg (reference threshold >400 mm Hg). During hospitalization, 36 (56%) cases had PaO_2_/FiO_2_ <200, and 12 (19%) cases had PaO_2_/FiO_2_ <100.

**Table 1:** Clinical characteristics of patients in study of severe YF, Brazil, 2017–2019*

Characteristic	Wild-type YF, n = 68	YEL-AVD, n = 5	Total YF, n = 73
Sex			
M	59 (86.8)	3 (60.0)	62 (84.9)
F	9 (13.2)	2 (40.0)	11 (15.1)
Age, y (range)	49 (35.5–60)	37 (32–49)	48 (34–60)
Previous medical condition			
Hypertension	19 (27.9)	2 (40.0)	21 (28.8)
Diabetes	8 (11.8)	0	8 (11.0)
Heart disease	5 (7.4)	0	5 (6.8)
Asthma/COPD	4 (5.9)	0	4 (5.5)
Habits			
Alcoholism	36 (52.9)	1 (20.0)	37 (50.7)
Smoking	26 (38.2)	1 (20.0)	27 (37.0)
Illicit drug use	11 (16.2)	0	11 (15.1)
Diagnostic criteria for YF			
Clinical criteria	68 (100)	5 (100)	73 (100)
Epidemiologic criteria	68 (100)	5 (100)	73 (100)
Live near to epizootic areas	46 (63)	NA	46 (63)
Travel to epizootic areas	35 (48)	NA	35 (48)
Serology, IgM, n = 47	37 (80)	NA	37 (80)
Time interval, d (range)			
Symptoms to hospitalization	4 (3–6)	5 (4–7)	4 (3–6)
Symptoms to death	9 (7–11)	8 (7–11)	9 (7–11)
Hospitalization to death	5 (3–6)	3 (2–5)	5 (3–6)
Intubation to death	2 (1–4)	2 (1–5)	2 (1–4)
Respiratory symptoms at admission			
Hemoptysis	6 (8.9)	1 (20)	7 (9.6)
Tachypnea	6 (8.9)	1 (20)	7 (9.6)
Cough	2 (3)	1 (20)	3 (4.1)
In-hospital events and interventions			
Shock or vasopressor use	68 (100)	5 (100)	73 (100)
Dialysis	58 (85.3)	4 (80.0)	62 (84.9)
Mechanical ventilation	68 (100)	5 (100)	73 (100)
Liver transplant for YF hepatitis	4 (5.9)	0	4 (5.5)
Secondary infection	53 (77.9)	3 (60.0)	56 (76.7)
Clinical diagnosis of pneumonia	5 (7)	0	5 (7)
SAPS 3, no. (range)	62 (31–104)	0	62 (31–104)
Vaccine status			
YF vaccine	9 (13.2)	5 (100)	14 (19.2)
Vaccination >10 d before symptoms	2 (2.9)	0	2 (2.7)
Laboratory tests, no. (range)	n = 64	n = 0	n = 64
Initial absolute neutrophil count, cells/µL	4,466 (2,433–6,633)		4,466 (2,433–6,633)
Initial lymphocyte count, cells/µL	675 (440–1,095)		675 (440–1,095)
Aspartate aminotransferase, U/L†	14,829 (3,523–75,000)		14,829 (3,523–75,000)
Alanine aminotransferase, U/L‡	6,074 (1,758–14,998)		6,074 (1,758–14,998)
Direct bilirubin, mg/dL§	7.26 (3.06–31.67)		7.26 (3.06–31.67)
Initial PaO_2_/FiO_2_ ratio	380 (281–471)		380 (281–471)
PaO_2_/FiO_2_ ratio during hospitalization, no. positive/no. tested (%)			
Moderate or severe, <200	36/64 (56)		36/64 (56)
Severe, <100	12/64 (19)		12/64 (19)

*Values are no. (%) except as indicated. COPD, chronic obstructive pulmonary disease; NA, not applicable; PaO_2_/FiO_2_, ratio of the partial pressure of oxygen in arterial blood to the inspired oxygen; SAPS 3, Simplified Acute Physiology Score III; YF, yellow fever; YEL-AVD, yellow fever vaccine–associated viscerotropic disease. †Reference range 10–40 U/L. ‡Reference range 4–36 U/L. §Reference value<0.3 mg/dL.

### Pathology

**Figure 1:**
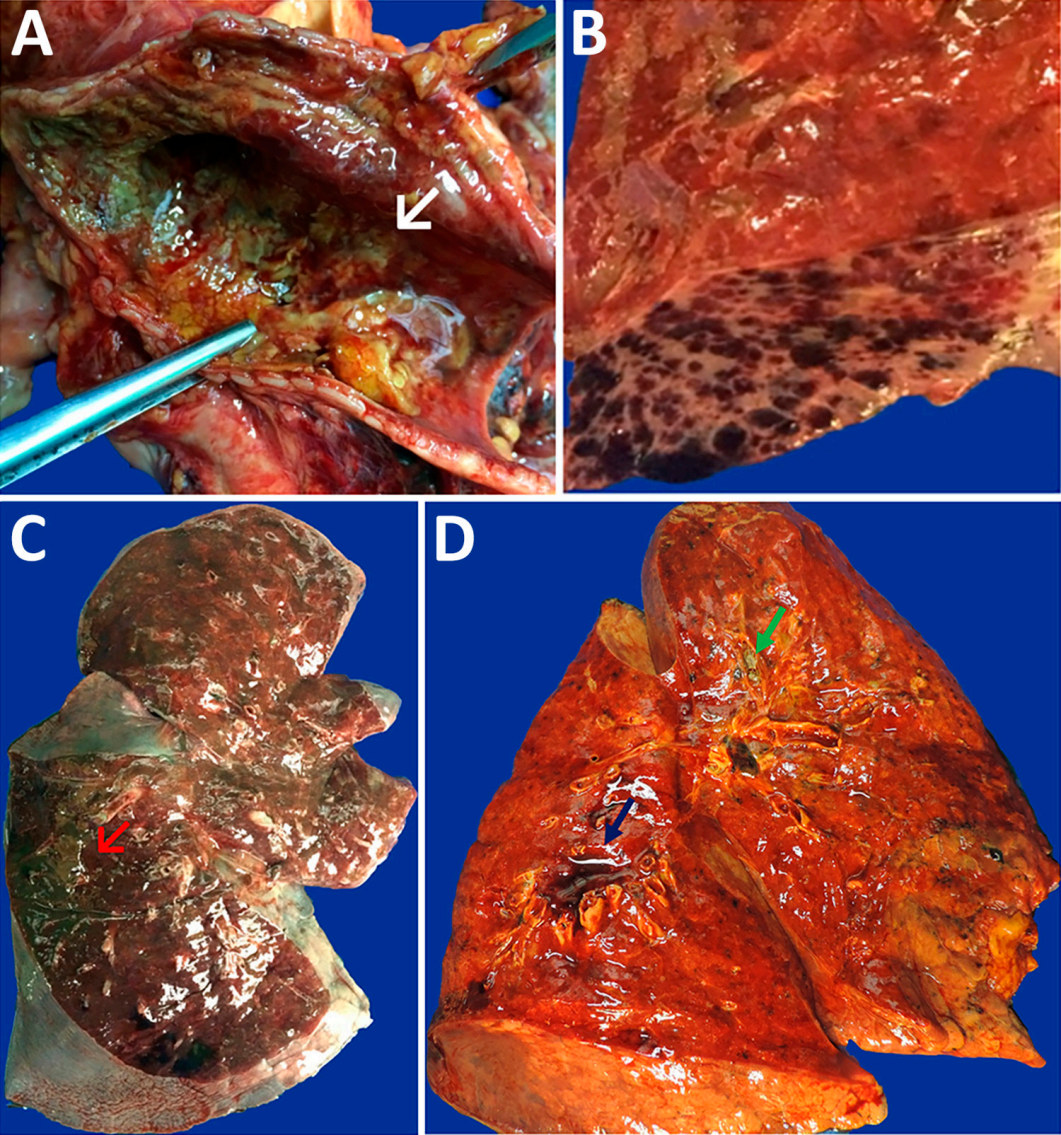
Macroscopic images of respiratory tract from patients with fatal yellow fever, São Paulo, Brazil, 2017–2019. A) Hemorrhagic necrosis of tracheal mucosa, covered with a thick whitish exudate (arrow), caused by *Candida* spp. invasive infection. B) Petechial pleural hemorrhage. C) Intense parenchymal edema and hemorrhage in the right lung, with massive gastrointestinal content aspiration in the posterior side (red arrow). D) Right lung with icterus, edema, hemorrhage, perivascular hemorrhage (blue arrow), and whitish exudate within bronchus (green arrow) caused by *Aspergillus* spp. infection.

All case-patients had visible signs of liver failure (jaundice, cavitary effusions, visceral congestion, edema, and hemorrhage) (Table 2). Gastrointestinal hemorrhages with signs of mesenteric ischemia and acute tubular necrosis were universal in variable degrees. The livers from all cases were steatotic with typical microscopic findings of YF hepatitis: midzonal hepatitis with steatotic and apoptotic hepatocytes, scarce inflammatory reaction, and positive expression of YFV antigens in degenerated hepatocytes and hepatic mononuclear cells. The main immediate cause of death was shock caused by hemorrhages in the gastrointestinal and respiratory tracts, refractory acidosis, and sepsis. At the respiratory tract, the main tracheal changes were congestion in 72 cases (99%), hemorrhage in 58 (79%), and ulceration in 41 (56%). Tracheal samples were available for 57 cases and showed tracheitis (91%), mucosal necrosis (45%), and microorganisms (35%) through histochemical stains. The lungs were heavier than normal; cut surfaces showed edema and hemorrhages, diffusely in both lungs, from all cases (Table 2). We observed friable cut surfaces mainly in middle and inferior pulmonary fields in cases with pneumonia (Figure 1). We noted macroscopic thrombi in 2 cases.

**Table 2:** Pulmonary findings in study of severe YF, Brazil, 2017–2019*

Characteristic	Wild-type YF, n = 68	YEL-AVD, n = 5	Total YF, n = 73
Pleural effusion			
Any effusion	18 (26)	2 (40)	20 (27)
Serous effusion	13 (19)	2 (40)	15 (21)
Hemorrhagic effusion	5 (7)	0	5 (7)
Volume, mL (range)	550 (400–1,000)	400 (300–500)	500 (400–950)
Pleuritis	38 (52)		38 (52)
Tracheal macroscopy			
Mucosal congestion	67 (99)	5 (100)	72 (99)
Mucosal edema	67 (99)	5 (100)	72 (99)
Mucosal ulceration	40 (59)	1 (20)	41 (56)
Mucosal hemorrhages	55 (81)	3 (60)	58 (79)
Tracheal histology	n = 54	n = 3	n = 57
Tracheitis	51 (94)	1 (33)	52 (91)
Squamous metaplasia	24 (44)	0	24 (42)
Necrotizing tracheitis	33 (61)	0	33 (45)
Infectious microorganism	19 (35)	1 (20)	20 (35)
Pulmonary macroscopic findings			
Right lung weight, g (range)†	758 (634–952)	752 (633–895)	755 (635–944)
Left lung weight, g (range)‡	658 (544–848)	534 (378–707)	654 (531–826)
Pulmonary cut surface with edema and hemorrhages	68 (100)	5 (100)	73 (100)
Friable pulmonary cut surface	59 (87)	2 (40)	61 (84)
Pulmonary microscopic findings			
Alveolar edema§	67 (99)	5 (100)	72 (99)
Mild	6 (9)	0	6 (8)
Moderate	36 (53)	1 (20)	37 (51)
Severe	25 (37)	4 (80)	29 (40)
Alveolar hemorrhage§	61 (90)	5 (100)	66 (90)
Mild	28 (41)	3 (60)	31 (42)
Moderate	25 (37)	1 (20)	26 (36)
Severe	8 (12)	1 (20)	9 (12)
Interstitial perivascular or peribronchial hemorrhage	51 (70)		51 (70)
Secondary pneumonia§	59 (87)	2 (40)	61 (84)
Mild	29 (43)	1 (20)	30 (41)
Moderate	17 (25)	0	17 (23)
Severe	13 (19)	1 (20)	14 (19)
Bilateral	47 (69)	2 (40)	49 (67)
Micro abscesses	22 (32)	1 (20)	23 (32)
Bronchoaspiration	42 (62)	2 (40)	44 (60)
Gram-positive cocci	5 (7)	1 (20)	6 (8)
Bacilli	55 (81)	4 (80)	59 (81)
Yeasts	23 (34)	4 (80)	27 (37)
Hyphae	8 (12)	1 (20)	9 (12)
Diffuse alveolar damage§	57 (84)	4 (80)	61 (84)
Mild	46 (68)	4 (80)	50 (68)
Moderate	11 (16)	0	11 (15)
Severe	0	0	0
Proliferative	4 (6)	0	4 (5)
Increased siderophages in the alveolar space	65 (96)	5 (100)	70 (96)
Increased megakaryocytes in the pulmonary parenchyma	62 (91)	5 (100)	67 (92)
Fibrin and fibrinoid endothelial necrosis	40 (59)	2 (40)	42 (58)
Pulmonary thrombi	22 (32)	1 (20)	23 (32)
Macroscopic thrombi	2	0	2
Microscopic thrombi	22	1	23 (32)
Hemophagocytosis¶	14 (21)	2 (40)	16 (22)
Bone marrow emboli	4 (5.5)	0	4 (5.5)
Emphysema	37	0	37 (50.7)
Foci of mononuclear perivascular infiltrate	68	5	73 (100)
Quantitative RT-PCR for YF	N = 61	N = 4	N = 65
Positive	57 (93)	4 (100)	61 (94)
Cycle threshold (range)	28 (26–32)	23 (22–32)	28 (26–32)

*Values are no. (%) except as indicated. RT-PCR, reverse transcription PCR; YF, yellow fever; YEL-AVD, yellow fever vaccine–associated viscerotropic disease. †Mean reference weight is 465 g. ‡Mean reference weight is 400 g. §Mild, present in 1%–25% of the histological sample; moderate, present in 25%–50%; severe, present in >50%. ¶Alveolar macrophages or in macrophages within the sinuses of intrapulmonary lymph nodes.

**Figure 2:**
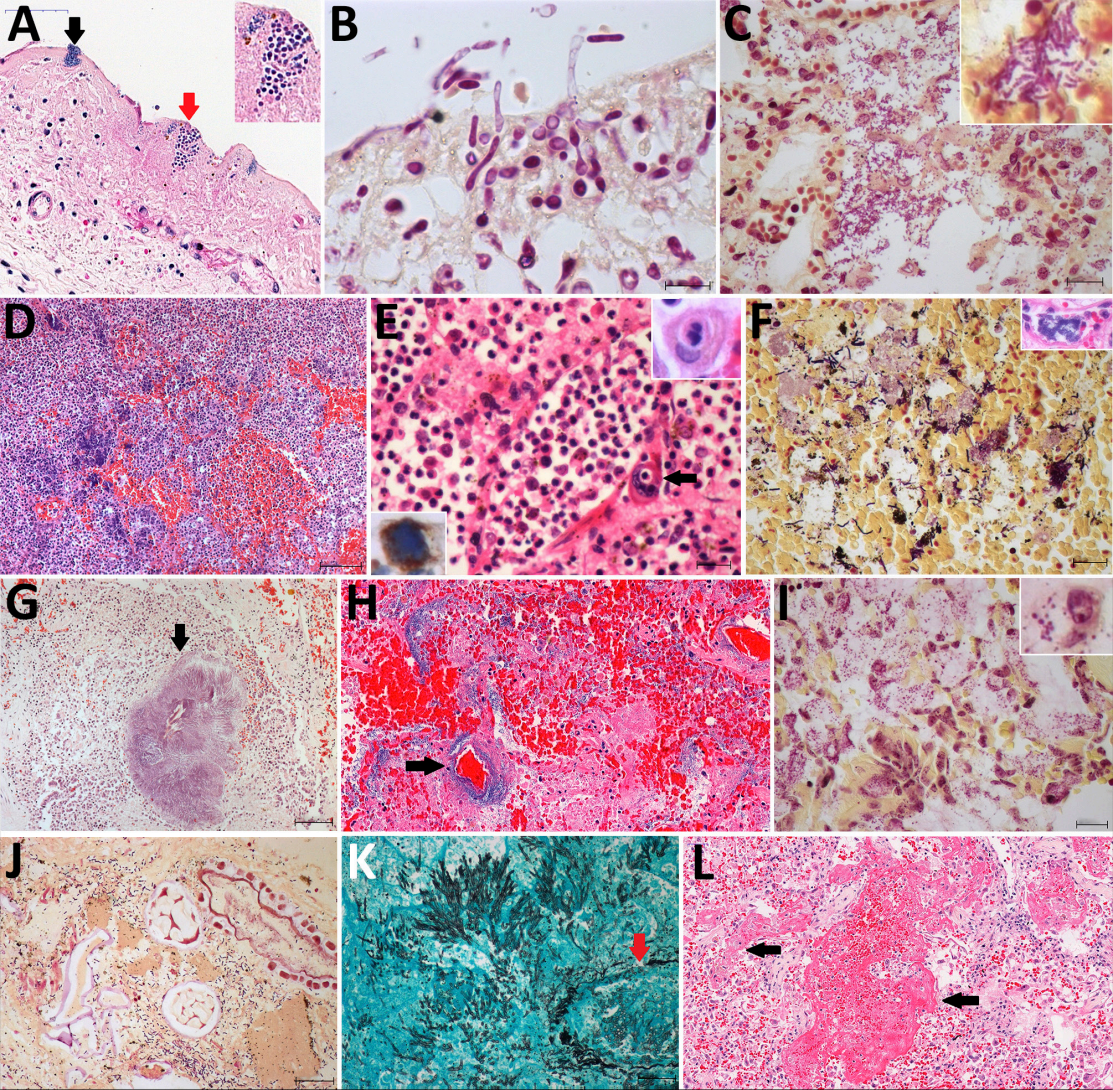
Pulmonary microscopic findings in patients with fatal yellow fever, São Paulo, Brazil, 2017–2019. A) Tracheal necrosis associated with bacilli (black arrow) and yeasts (inset and red arrow). Hematoxylin and eosin (HE) stain; scale bar = 50 µm. B) *Candida* *albicans* pseudohyphae and hyphae invading necrotic tracheal mucosa. Gram stain in immersion oil; scale bar = 10 µm. C) Bronchopneumonia associated with gram-negative bacilli. Gram stain; scale bar = 20 µm. D) Hemorrhagic pneumonia with microabscess composed of macrophages, neutrophils and colonies of coccus. HE stain; scale bar = 100 µm. E) Suppurative pneumonia showing hemophagocytosis (right inset) and a megakaryocyte in a septal vessel (arrow), with emperipolesis. HE stain; scale bar = 20 µm. Left inset: megakaryocyte labeled by VIII factor antigen detected by immunohistochemistry. Peroxidase stain. F) Polymicrobial aspirative pneumonia with gram-positive cocci and gram-positive and gram-negative bacilli with different morphologies; the inset shows a colony of bacilli in a septal vessel corresponding to agonal bacteremia. Gram stain; scale bar = 20 µm. Inset: HE stain. G) *Actynomyces* granule (arrow) with degenerated squamous cells in the center in an area of aspirative pneumonia. HE stain; scale bar = 100 µm. H) *Pseudomonas* hemorrhagic pneumonia, with numerous bacilli surrounding a septal vessel (arrow). HE stain; scale bar = 50 µm. I) *Mycoplasma salivarium* pneumonia, showing tiny gram-negative bacilli (inset) in the cytoplasm of intrabronchial macrophages. Gram stain; scale bar = 20 µm. J) Bronchoaspiration of vegetal alimentary material, associated with gram-negative bacilli, in the alveolar space. Gram stain; scale bar = 20 µm. K) Pulmonary angioinvasive aspergillosis, with typical hyphae invading pulmonary vessel (arrow), with associated necrosis and mild neutrophilic reaction. Grocott-Gomori methenamine silver stain; scale bar = 20 µm. L) Exudative diffuse alveolar damage, with congestion, alveolar edema, and hyaline membranes (arrow). HE stain; scale bar = 100 µm. Insets: original magnification ×400.

**Figure 3:**
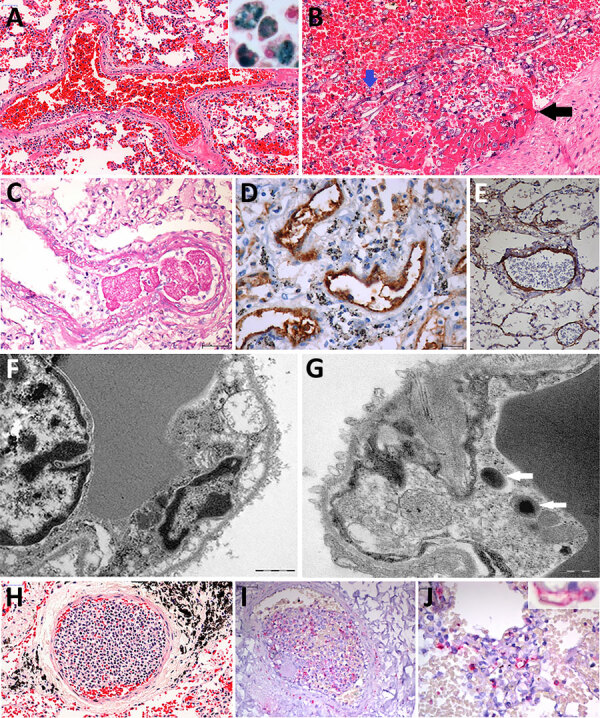
Pulmonary vascular damage in fatal yellow fever cases, 2017–2019 epidemic, São Paulo, Brazil. A) Medium-sized artery with fibrinoid necrosis of the endothelial layer, marginated leukocytes, wall edema, septal congestion, alveolar hemorrhage. Hematoxylin and eosin (HE) stain; scale bar 50 µm. Inset shows group of hemosiderin-laden alveolar macrophages stained for iron. Perls stain; original magnification ´200. B) Pulmonary artery showing angioinvasion by *Aspergillus* spp. forming fibrinous thrombus on the endothelial vascular layer (black arrow). HE stain; scale bar = 50 µm. C) A small fibrin clot and the artery fibrinoid necrosis and wall edema. Periodic acid–Schiff stain; scale bar = 50 µm. D) Positive detection of VIII coagulation factor in the entire wall of pulmonary arteries. Peroxidase stain; scale bar = 20 µm. E) The VCAM is detected in the endothelial and muscular pulmonary artery layers. Peroxidase stain; scale bar = 20 µm. F) Septal capillaries showing mitochondrial dilation with loss of cristae. Ultrathin section; scale bar = 1 µm. G) Bacilli (arrows) within septal pulmonary vessel. Ultrathin section; scale bar = 500 nm. H) Histologic leukostasis in a septal pulmonary artery, showing immature myeloid cells, lymphocytes, and neutrophils. HE stain; scale bar = 50 µm. I) Intravascular cells expressing yellow fever virus antigens in their cytoplasm. Alkaline phosphatase stain; scale bar = 50 µm. J) The yellow fever virus antigen is detected in the cytoplasm of septal endothelial cells (inset; original magnification ´400) and in interstitial and alveolar inflammatory cells. Alkaline phosphatase stain; scale bar = 20 µm.

The main microscopic findings were alveolar edema in 72 (99%) cases, alveolar hemorrhage in 66 (90%) cases, and secondary suppurative pneumonia in 61 (84%) cases; pneumonia was bilateral in 49 (67%) cases, with microabscesses in 23 (32%) cases, and associated with signs of bronchoaspiration (alimentary vegetal material in pulmonary tissue) in 44 (60%) cases (Figure 2). Gram-negative bacilli were detectable in 59 (81%) cases, yeasts in 27 (37%) cases, hyphomycetes in 9 (12%) cases, and gram-positive cocci in 6 (8%) cases. The yeasts had morphologic aspects compatible with *Candida* spp.; most hyphae had morphology compatible with *Aspergillus* spp. One case had hyphae resembling *Mucorales* spp. Fungal pneumonia was associated with angioinvasion, tissue necrosis, cellular debris, and scarce inflammatory reaction surrounding the fungal forms. We found septic pulmonary vasculitis in 13 (21.3%) cases: in 1 (7.7%) case from gram-positive cocci, in 2 (15.3%) from gram-negative bacilli, in 2 (15.3%) from yeasts, and in 8 (80%) from hyphomycetes (Figures 2, 3). Septal capillaries showed increased numbers of circulating megakaryocytes in 67 (92%) cases and fibrinoid necrosis in 42 (58%) cases. We observed microscopic thrombi in 23 (32%) cases and diffuse alveolar damage (DAD) in 61 (84%) cases, mainly in the exudative phase (Figure 2). All patients with PaO_2_/FiO_2_ ratio <300 had DAD. We observed hemophagocytosis in the intraalveolar macrophages or the intrapulmonary lymph nodes in 16 (22%) cases. One case showed a calcified primary complex at the hilum and another an isolated cryptococcoma. We observed no viral cytopathic effect. 

All cases had an increased expression of VIII coagulation factor and VCAM in the pulmonary vessels (Figure 3). All 3 cases examined by EM of the lung tissue showed altered endothelial cells cytoplasmatic pseudopods in the pulmonary septa, intravascular fibrin, and intraalveolar or intravascular bacilli (Figure 3). YFV particles were not found. All cases had positive YFV antigens in pulmonary tissue, staining the cytoplasm of scattered endothelial cells from septal capillaries and interstitial inflammatory cells (Figure 3).

### Molecular Analysis in Fresh Frozen Lung Tissue

We detected YFV RNA in 61 (94%) cases of the lung tissue samples analyzed. In addition, Sanger sequencing of 48 tissue samples enabled us to identify secondary bacterial and fungal infections in samples collected during autopsy (Table 3; Table 4; Table 5). The sequences obtained in this study have been deposited in GenBank (Table 3; Table 4; Table 5). Bacterial infections were detected in 41 cases (85.4%), fungal infections in 21 cases (43.7%), and mixed infections in 14 cases (29%). The bacterial genera most frequently associated with pneumonia in those 41 cases were *Enhydrobacter* in 13 (31.7%) cases, *Klebsiella* in 9 (22%) cases, *Acinetobacter* in 5 (12.3%) cases, and *Pseudomonas* in 3 (7.3%) cases (Appendix Table). The most prevalent fungal genera in those 21 cases were *Candida* in 10 (47.6%) cases, *Aspergillus* in 4 (19%), and *Trichosporon* in 2 (9.5%). We were unable to detect the presence of microorganisms by nested PCR in 25 cases. Among 48 PCR-positive cases, histological correlation was absent in 1 (*Cladosporium sphaerospermum*, not visualized in a case with pneumonia). In 47 cases, PCR findings matched histopathology: 43 with complete correlation, 3 with partial correlation (>1 agent identified within pneumonia), and 1 case corresponding to terminal aspiration rather than pneumonia.

**Table 3:** Characteristics of yellow fever cases with bacterial infections in the lung, Brazil, 2017–2019*

Pathogen	GenBank accession no.	YFV type	Gram stain
*Enhydrobacter aerosaccus*	MN436791	Sylvatic	Negative
	MN439924	Sylvatic	Negative
	MN44786	Sylvatic	Negative
	MN447222	Sylvatic	Negative
	MN447318	Sylvatic	Negative
	MN447732	Sylvatic	Negative
	MN431431	Sylvatic	Negative
	MN441757	Negative	Negative
	MN44855	Sylvatic	Negative
	MN445607	Vaccinal	Negative
	MN447234	Sylvatic	Negative
	MN447305	Sylvatic	Negative
	MN447407	Sylvatic	Negative
*Klebsiella pneumoniae*	MN437654	Sylvatic	Negative
	MN44823	Sylvatic	Negative
	MN447127	Sylvatic	Negative
	MN526933	Sylvatic	Negative
	MN447416	Sylvatic	Negative
	MN447588	Sylvatic	Negative
	MN447652	Sylvatic	Negative
	MN447666	Sylvatic	Negative
	MN442076	Vaccinal	Negative
*Acinetobacter baumannii*	MN428414	Sylvatic	Negative
	MN435153	Sylvatic	Negative
	MN431185	Sylvatic	Negative
	MN447636	Sylvatic	Negative
*Acinetobacter* sp.	MN445371	Sylvatic	Negative
*Pseudomonas aeruginosa*	MN429315	Sylvatic	Negative
*P. baetica*	MN447128	Sylvatic	Negative
	MN447668	Sylvatic	Negative
*Escherichia coli*	MN436844	Sylvatic	Negative
	MN437322	Sylvatic	Negative
*Mycoplasma salivarium*	MN447214	Sylvatic	
*M. orale*	MN447304	Sylvatic	
*Moraxella osloensis *	MN445979	Vaccinal	Negative
	MN437656	Sylvatic	Negative
*Streptococcus pneumoniae *	MN431202	Sylvatic	Positive
*Serratia marcescens*	MN431240	Sylvatic	Negative
*Cronobacter dublinensis*	MN431459	Sylvatic	Negative
*Simiduia agarivorans*	MN447394	Sylvatic	Negative
*Enterobacter asburiae*	MN430860	Sylvatic	Negative

*A total of 41 bacterial infections represented 56.75% of study cases. YFV, yellow fever virus.

**Table 4:** Characteristics of 21 yellow fever cases with fungal infections in the lung, Brazil, 2017–2019*

Pathogen	GenBank accession no.	YFV type
*Nakaseomyces glabratus*†	MN473880	Sylvatic
	MN475751	Sylvatic
	MN477933	Sylvatic
	MN475749	Sylvatic
*Candida albicans*	MN473077	Sylvatic
	MN475275	Sylvatic
	MN477032	Sylvatic
*C. tropicalis*	MN477465	Sylvatic
	MN475173	Vaccinal
*C. parapsilosis*	MN477247	Sylvatic
*Aspergillus fumigatus*	MN474008	Sylvatic
	MN475197	Sylvatic
	MN477796	Sylvatic
*A. flavus*	MN477210	Sylvatic
*Trichosporon faecale*	MN472741	Sylvatic
*T. asahii*	MN475174	Vaccinal
*Didymella glomerata*	MN472927	Sylvatic
	MN472744	Vaccinal
*Debaryomyces hansenii*	MN472906	Sylvatic
*Cladosporium sphaerospermum*	MN476933	Sylvatic
*Apiotrichum domesticum*	MN477197	Sylvatic

*A total of 21 fungal infections represented 28.4% of study cases. YFV, yellow fever virus. †Formerly known as *Candida glabrata*.

**Table 5:** Cases of severe yellow fever with bacterial and fungal co-infections in the same case, detected in the lung tissue by nested RT-PCR and genomic sequencing, Brazil, 2017–2019*

Case no.	YFV type	Bacteria				Fungus	
		Organism	GenBank accession no.	Gram stain		Organism	GenBank accession no.
1	Sylvatic	*Pseudomonas aeruginosa*	MN429315	Negative		*Nakaseomyces glabratus*†	MN473880
2	Sylvatic	*Streptococcus pneumoniae*	MN431202	Positive		*Debaryomyces hansenii*	MN472906
3	Sylvatic	*Enhydrobacter aerosaccus*	MN44786	Negative		*Aspergillus fumigatus*	MN474008
4	Sylvatic	*P. aeruginosa*	MN447128	Negative		*A. fumigatus*	MN475197
5	Sylvatic	*Mycoplasma salivarium*	MN447214			*Candida albicans*	MN475275
6	Sylvatic	*Klebsiella pneumoniae*	MN447588	Negative		*Apiotrichum domesticum*	MN477197
7	Sylvatic	*K. pneumoniae*	MN447652	Negative		*N. glabratus*†	MN477933
8	Sylvatic	*Enterobacter asburiae*	MN430860	Negative		*Trichosporon faecale*	MN472741
9	Vaccinal	*Moraxella osloensis*	MN445979	Negative		*Trichosporon asahii*	MN475174
10	Vaccinal	*E. aerosaccus*	MN445607	Negative		*Candida tropicalis*	MN475173
11	Sylvatic	*E. aerosaccus*	MN447234	Negative		*N. glabratus*†	MN475749
12	Sylvatic	*E. aerosaccus*	MN447407	Negative		*C. albicans*	MN477032
13	Sylvatic	*Acinetobacter baumannii*	MN447636	Negative		*Candida parapsilosis*	MN477247
14	Sylvatic	*K. pneumoniae*	MN447416	Negative		*A. fumigatus*	MN477796

*YFV, yellow fever virus †Formerly *Candida glabrata*.

## Discussion

In this study, we describe results of the autopsies of patients who died of severe YF, focusing on pathologic findings in the respiratory tract. The classic pulmonary findings in YF (*8*,*20*), such as edema and alveolar hemorrhage, were very frequent as expected; however, we also observed a high incidence (84%) of extensive pneumonia caused by bacterial and fungal microorganisms, associated with exudative diffuse alveolar damage, which certainly contributed to the fatal outcomes. We also discovered an unexpected and frequent finding of infectious necrotizing tracheitis, which supports the rationale that inhalation or aspiration are the main routes of respiratory infection in those cases. Of note, few patients (7%) had clinical suspicion of pneumonia during their intensive care stay. Some of them had a clinical diagnosis of sepsis, albeit with a poorly defined site, mainly based on persistent fever, clinical deterioration, and progressive neutrophilia with left deviation and elevated serum C-reactive protein. The difficulty in determining the primary site of infection was multifactorial: rapid clinical progression and hemorrhagic phenomena, making diagnostic procedures such as bronchoalveolar lavage and chest tomography difficult; use of broad-spectrum antimicrobial drugs as prophylaxis for infections associated with acute liver failure, rendering cultures negative; pulmonary hemorrhage masking new infiltrates associated with pneumonia on chest radiographs taken in bed; misattribution of septic signs to YFV infection; and finally, little understanding of the disease, which was previously uncommon in urban centers in southeastern Brazil (*1*–*6*).

The respiratory secondary infections we described are not usually reported in YF autopsies, although they are a common cause of infection and death in patients with acute liver failure of other etiologies (*20*–*25*). Our data are the result of thorough macroscopic examination in the autopsy room and extensive lung sampling for microscopy and molecular analysis. In addition, the minimally invasive tissue sampling protocol we used during the 2017–2019 YF epidemic was also successful in diagnosing pneumonia at postmortem examination in these cases (*9*).

The pattern of tracheitis and pneumonia observed at autopsy shows aspirative, nosocomial, and opportunistic elements (*25*,*26*). In the pathogenesis of the aspirative and nosocomial elements, massive gram-negative bacilli (including nonfermenting bacilli and Enterobacteriaceae) from the gastrointestinal flora colonized and infected the upper and lower airways of patients with severe YF. The uncontrollable vomiting associated with hepatic coma and gastrointestinal bleeding and ischemia might have caused bronchoaspiration. Other predisposing factors are the presence of an endotracheal tube and mechanical ventilation and the selective pressure of broad-spectrum antimicrobial drugs prescribed for prophylaxis in fulminant hepatitis for opportunistic nonfermenting gram-negative bacilli (e.g., *Enhydrobacter*, *Klebsiella*, *Acinetobacter*, and *Pseudomonas*) and non-*albicans* strains of *Candida* spp. The presence of the endotracheal tube might have caused the unusual finding of ulcerative tracheitis; however, our intensive care unit staff follows standard protocols to avoid endotracheal tube–related injuries, which are also rare in our autopsy routine (*7*,*11*,*13*,*25*,*26*).

Another element that we highlighted was an intense and systemic immune dysfunction in severe YF cases, which probably also lowered the immunity of the respiratory mucosal barrier, predisposing case-patients to invasive infections by bacteria and fungi, including *Aspergillus* spp., commonly described in severely lymphopenic or neutropenic patients. That opportunistic element is supported by evidence: peripheral lymphopenia (Table 1), an increase in inflammatory cytokines similar to that seen in patients with lethal bacterial sepsis, lymphoid depletion, and hemophagocytosis in secondary lymphoid organs, including the intrapulmonary lymph nodes, spleen, and bone marrow (*7*,*9*,*10*,*17*,*18*).

Disseminated angioinvasive infections by hyphomycetes in cases with severe YF, involving the lungs and other organs, have been reported previously in humans and neotropical nonhuman primates, which suggests a direct effect of YFV on lymphoid organs regardless of the host and independent of intensive treatment (*21*,*22*,*27*). Further analysis focusing on the airway mucosal immunity would clarify some gaps in the host immune response in severe YF.

We detected YFV RNA in 94% of the lung samples by RT-PCR and YFV antigens in all cases by immunohistochemistry in endothelial and inflammatory cells. Those findings corroborate that YFV induces systemic vascular damage associated with hemorrhagic phenomena (*2*,*7*,*8*). It is possible that pulmonary edema and hemorrhage represent the alterations most directly associated with YFV-induced vasculopathy. However, we propose the extensive pneumonia observed in our cases, with consequent sepsis, exacerbates the vascular damage initiated by the YFV. Moreover, pneumonia induces exudative diffuse alveolar damage, leading to hypoxemia and consequent respiratory failure, adding more vascular damage. One striking finding was thrombosis in the pulmonary vasculature, which appeared to be recent and have little organization on histology, mainly affecting small and medium-sized vessels. We observed macroscopic thrombi in 2 cases, suggesting that thrombosis is mainly formed in the intrapulmonary bed, triggered by intense inflammation in the lung tissue, rather than by thromboembolism. Some cases also had fungal angioinvasion and septic vasculitis (Table 2; Figures 2, 3). We observed a complex coagulopathy in fatal cases of YF. Higher levels of D-dimer in those cases than in survivors did not seem to be related only to liver failure, as observed in an experimental model of YFV infection, which suggests a concurrent consumptive coagulopathy in severe YF (*28*). Pulmonary vascular thrombosis could explain the increase in serum D-dimer; other possible sources are intestinal ischemia and hemophagocytes, which should also be investigated in severe YF (*9*,*17**,**29*).

We found a similar pattern of secondary infections, vasculopathy, and evidence of direct YFV damage in hearts from the same YF autopsy cohort (*18*). We also observed in the hearts from YF case-patients an increased in situ expression of inflammatory cytokines and markers of endothelial damage, similar to those who died of bacterial sepsis and septic shock (*18*). Our results support the concept that the period of intoxication is characterized by sepsis in those with a fatal outcome.

**Figure 4:**
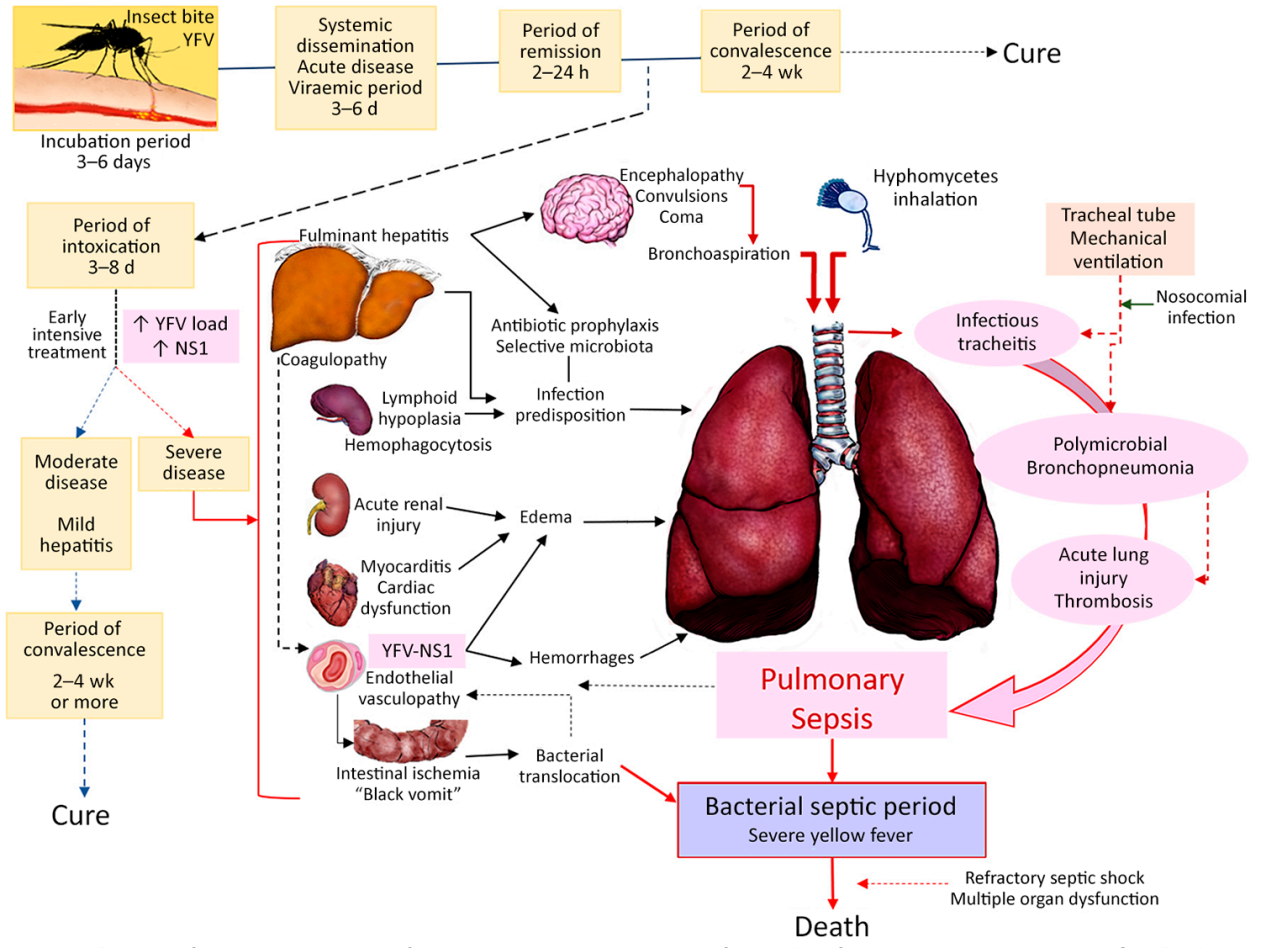
Proposal for the pathogenesis of respiratory tract involvement in fatal yellow fever, 2017–2019, epidemic, São Paulo, Brazil. After the mosquito bite with inoculation of YFV virions into the skin, there is a systemic spread of YFV with viremia that lasts for 5–7 days. The virus replicates in the blood, liver, spleen, kidneys, heart, brain, lungs, and other internal organs, and patients have nonspecific viral symptoms. After that is the toxemic phase, which is more severe in those with higher YFV-RNA load and higher YFV-NS1, with hepatitis, acute renal injury, immune dysfunction (lymphoid hypoplasia and hemophagocytosis), myocarditis, and systemic vascular damage. The lungs are affected in a multifactorial way. The acute liver failure due to YF-fulminant hepatitis and immune dysfunction predisposes to secondary infection. In particular, hepatic encephalopathy leads to aspiration of gastrointestinal microbiota (bacilli and *Candida* spp.) or inhalation of ubiquitous filamentous fungi that cause polymicrobial pneumonia and acute lung injury. The tracheal tube exerts mechanical damage to the tracheal mucosa, producing acute tracheitis associated with the aspirated flora, with ulceration and mucosal necrosis. Acute renal and myocardial dysfunction by YFV contribute to pulmonary edema. The endothelial injury also contributes to pulmonary edema which, in association with liver failure coagulopathy, leads to pulmonary hemorrhage. Acute lung injury caused by pneumonia, edema, and hemorrhage plus intestinal bacterial translocation in severe cases, presumed by evidence of intestinal ischemia and severe liver damage at autopsy, lead to bacterial sepsis, which amplifies the initial endothelial dysfunction generated by YFV, causing refractory shock and subsequent death. NS, nonstructural protein; YFV, yellow fever virus.

Our results are somewhat related to those of a previous study in the city of São Paulo during the same epidemic, which showed that, in 76 patients with symptomatic YF, markers of death included neutrophilia >4,000 cells/mL, older age, high aspartate transaminase, and high viral load at hospital admission (*10*). Other studies from the clinical cohort of YF cases have shown that YFV proteins nonstructural 1, syndecan 1, and angiopoietin 2 were higher at hospital admission in patients with a fatal outcome than in those who survived and in normal controls without heart infection (*15*,*16*). Those markers were also high in the hearts of the cases in our study but similar to those observed in sepsis (*18*). Of note, our autopsy series includes most of the fatal cases reported in our clinical cohort study and in other clinical studies from the 2017–2019 YF epidemics in São Paulo (*7*,*11*,*13*). We believe that those markers reflect not only an intense viral infection with extensive liver necrosis but also an ongoing sepsis originating from the respiratory tract and also from bacterial translocation associated with intestinal ischemia that aggravates the systemic inflammatory response and endothelial damage induced by YFV and determines the fatal fate of some patients during the period of intoxication (*29*). All those processes are interconnected in a complex way (Figure 4). 

A limitation of this study is that few pathogens were recovered in culture before death, and therefore, little correlation can be made with histologic or molecular postmortem results. However, we saw great correlation between the PCR results and the histopathologic study with ancillary histochemical stains in our study. We performed the treatments and postmortem examinations within a single institution, which ensured consistency of results and standardized clinical and pathologic protocols. Nonetheless, the single-site nature of the study might limit the generalizability of our findings to resource-limited settings where YF is endemic.

In conclusion, severe YF is a complex, systemic viral disease that affects other organs in addition to the liver. The respiratory tract is affected both by YFV, as evidenced by the detection of viral antigens and RNA in the lung parenchyma, and by secondary infections (tracheitis and pneumonia) of a multifactorial nature, which amplify the initial systemic inflammatory response, endothelial damage, and coagulopathy caused by YFV. Secondary sepsis and DAD exacerbate the intoxication phase of YF and lead patients to unfavorable outcomes. Our findings could guide the management of severe YF and help to establish preventive measures for bronchoaspiration and for the diagnosis and treatment of secondary respiratory infections. This study demonstrates the role of autopsy in better understanding the pathogenesis of infectious diseases during epidemics, especially at a time when arboviruses are reemerging, as seen in Brazil in 2024 with the catastrophic dengue epidemic and the spread of Oropouche fever and in 2025 with a new epidemic of YF in the state of São Paulo (*30*,*31*).

Appendix Additional information about pulmonary complications in fatal yellow fever, Brazil, 2017–2019.
