# Supplementary material for: Pulmonary Complications in Fatal Yellow Fever, Brazil, 2017–2019
Source: Emerg Infect Dis. 2026 Feb;32(2):211–24. doi: 10.3201/eid3202.250530 (PMC12928214; doi:10.3201/eid3202.250530)
Supplement: Appendix — Additional information about pulmonary complications in fatal yellow fever, Brazil, 2017–2019. [file 25-0530-Techapp-s1.pdf]

Article DOI: https://doi.org/10.3201/eid3202.250530

EID cannot ensure accessibility for supplementary materials supplied by authors. Readers who have difficulty accessing supplementary content should contact the authors for assistance.

Pathogenesis of Pulmonary Complications in Fatal Yellow Fever, Brazil, 2017–2019

Appendix

Methods

Subjects, Study Settings and Autopsy Protocol

The cases with wild-type/sylvatic YF were infected in cities north of the metropolitan region of São Paulo or on the northern coast of the state, mainly Mairiporã, Cotia, Atibaia, Serra da Cantareira, Caraguatatuba, and also Arujá, Aguaí, Ibiúna, and Santa Izabel (*1*–*5*). Three cases were infected in the state of Minas Gerais (Ponte Nova, Machado, and Itambacuri).

Patients were not using immunosuppressants, including corticosteroids, and were tested for HIV upon admission, all negative. Other causes of hepatic failure were excluded: Hepatitis A, B, E, D, dengue, zika, chikungunya, Cytomegalovirus, adenovirus, HSV-1 and 2 (serology and PCR), and toxic/alcoholic hepatitis (medical history from relatives).

Immunohistochemistry

To validate the anti-YFV antibody, liver samples from YFV-confirmed cases were used as positive controls, with high viral load in the blood and in the liver tissue. Negative controls included liver biopsy and autopsy samples from patients with other infectious diseases, such as dengue, oropouche fever, hepatitis B virus (HBV), hepatitis A virus (HAV), hepatitis C virus (HCV), human herpesvirus 6 (HHV-6), human herpesvirus 8 (HHV-8), Epstein-Barr virus (EBV), herpes simplex virus 1 (HSV-1) and 2 (HSV-2), *Mycobacterium avium*, leptospirosis, and rickettsiosis. We also tested the primary antibody in normal lung samples and in samples from patients with influenza H1N1 pneumonia, measles, respiratory syncytial virus, herpes virus, cytomegalovirus, parainfluenza virus, monkeypox virus, and adenovirus. A cerebral sample infected with Zika virus, obtained from our autopsy archive, was also tested. The IHC reaction

was negative in all of these samples, as well as in samples tested with the isotype and with suppression of the primary anti-YFV antibody.

Viral RNA Detection

We executed quantitative RT-PCR (RT-qPCR) for YF viral RNA in fresh frozen tissues collected from patients. Fragments measuring 1 cm^3 were first macerated, then nucleic acid were extracted using guanidine thiocyanate (TRIzol, Life Technologies). Appropriate reagents for RT-qPCR were then used (AgPath-ID one-step RT–PCR reagents, Ambion) with primers and probes described in Appendix Table. RT-qPCR reactions consisted of a reverse transcription step, at 45 C for 10 min plus 95 C for 10 min for enzyme activation, and 40 cycles at 95 C for 15s plus 60 C for 45s for hybridization and extension, using the ABI7500 equipment (Thermo Fisher Scientific).

DNA Extraction—Fungal and Bacterium

To extract DNA from lung tissue, 100µ samples were mixed with 500µL ATL buffer (QIAamp DNA Mini-kit, Qiagen, Hilden, Germany) for 3h at 56°C with 100 mg/mL proteinase K (Qiagen, Hilden, Germany) in thermomix (Thermomixer compact – Eppendorf). DNA was then extracted using Magna Pure Compact Nucleic Acid Isolation kit (Magna Pure Compact Roche, Penzberg, Germany).

PCR

The presence of amplifiable DNA was confirmed by nested PCR of a fragment of human glyceraldehyde-3-phosphate dehydrogenase (GADPH; GenBank: J04038.1), as described previously (*6*). Outer primers 5′ GAC AAC AGC CTC AAC ATC ATC 3′ and 5′ GAC GGC AGG TCA GGT CCA CCA 3′ were used to amplify a 610 bp fragment, and inner primers 5′AAT GCC TCC TGC ACC ACC 3′ and 5′ ATG CCA GTG AGC TTC CCG 3′ were then used to amplify an internal 248bp product. In the first round, targets were amplified from 2µL DNA extract in 25µL of 10mM Tris-HCl pH 8.3, 50mM KCl, 2.5mM MgCl2, 0.3µM each of outer primers, 1.5U AmpliTaq DNA polymerase, and 100µM of each dNTP, over one cycle at 94°C for 5 min, 35 cycles at 94°C for 30s, 56°C for 30s, and 72°C for 45s, and final extension at 72°C for 5 min. In the second round, targets were amplified from 2µL of the initial amplification product in 50µM of each dNTP and 0.3µM of each inner primer, over the same thermal profile as

the first reaction, except that 40 cycles were carried out. Positive and negative controls without DNA were included in all assays.

Fungal -Nested PCR 5.8SrDNA

For nested PCR of 5.8SrDNA-ITS, all strains were first sequenced with outer primers its1 - 5′ TCC GTA GGT GAA CCT GCG G 3′ and its4 - 5′ TCC TCC GCT TAT TGA TAT GC 3′, were used to amplify a 600–650bp sequence from several fungi that are pathogenic to humans. Inner primers, its3 - 5′ GCA TCG ATG AAG AAC GCA GC 3′, and its4 - 5′ TCC TCC GCT TAT TGA TAT GC 3′ were used to amplify na internal 250–432bp product, to target the conserved 18S, 5.8S, and 28S regions of the rRNA gene (*7*).

In the first round, its1, its3 and its4 primers were then used to amplify the intervening HC5.8S-ITS sequence in 25 µL as described previously (*7*), using conditions described in Fujita et al. (*8*). In the primary round, reactions consisted of 2 µL DNA extract in 10 mM Tris-HCl pH 8.3, 50 mM KCl, 2.5 mM MgCl2, 1 µM outer primers, 1.5 U Platinum Taq DNA poly Brazil, and 100 µMeach of dNTP. Targets were amplified over one cycle at 94°C for 5 min, 35 cycles at 94°C for 30 s, 65°C for 30 s, and 72°C for 1 min, and one cycle at 72°C for 5 min. Reaction mixtures in the second round were identical, except that 2 µL of the first reaction product, 50 µM dNTP, and 1 µM each of inner primers were used. Positive and negative controls without DNA were included in all assays.

Bacteria- PCR 16SrDNA

For PCR of 16S rDNA, all strains were sequenced with outer primer, BAC-F 5′-TCCTACGGGAGGCAGCAGT-3′ and BAC-R 5′-GGACTACCAGGGTATCTAATCCTGTT-3′ were used to amplify a 466bp sequence from several bacteria that are pathogenic to humans The presence of amplifiable DNA was confirmed by PCR as described previously 16SrDNA (*9,10*). The reaction consisted of 2µL DNA extract in 10mM Tris-HCl pH 8.3, 50 mM KCl, 2.5mM MgCl2, 1µM primers, 1.5U Platinum Taq DNA poly Brazil, and 100 µM each of dNTP. Targets were amplified over one cycle at 94°C for 5 min, 35 cycles at 94°C for 30 s, 65°C for 30 s, and 72°C for 1 min, and one cycle at 72°C for 5 min. Positive and negative controls without DNA were included in all assays.

All PCR reagents were obtained from Invitrogen (Carlsbad, CA, USA), and samples were processed and amplified three times on a Veriti 96 thermocycler (Applied Biosystems, Life Technologies Corporation, Carlsbad, CA, USA). To avoid contamination of components, preparation of reaction mixtures and addition of template DNA were performed in separate rooms. All assays included negative controls without DNA and positive controls

For fungal strains control (DNA) - *H. capsulatum* ATCC A811 and B923, *C. neoformans* ATCC 24067, *P. brasiliensis* 18 and 339, *Aspergillus* spp, *Candida albicans* and *C. parapsilosis* strains were available from previous studies.

For bacterium strains control (DNA) - *Escherichia coli* (JM109) and *Pseudomonas aeruginosa* (ATCC 15442) strains were available from previous studies.

Products were electrophoresed on 1.5% agarose, stained with ethidium bromide, and visualized on a UV transilluminator and PCR products were purified with PureLink Kit (QIAGEN).

Sequencing

Samples of lung tissue in which fungal and bacterium was detected by PCR were submitted to sequencing analysis according to the method of Sanger. The sequencing reaction was performed using the kit ABI PRISM BigDye Terminator Cycle Sequencing Ready Reaction (Applied Biosystems, Life Technologies, Carlsbad, California, United States) according to the manufacturer's instructions. Sequencing was completed using the Genetic Analyzer ABI 3130 (Applied Biosystems, Life Technologies, Carlsbad, California, U.S).

The sequences were analyzed using the program Sequencher 3.0 Sequencing Software (Ann Arbor, Michigan, United States) and compared with the genomic database GenBank (National Center for Biotechnology Information—NCBI- Bethesda, Maryland, U.S).

Results

References

1. Possas C, Lourenço-de-Oliveira R, Tauil PL, Pinheiro FP, Pissinatti A, Cunha RVD, et al. Yellow fever outbreak in Brazil: the puzzle of rapid viral spread and challenges for immunisation. Mem Inst Oswaldo Cruz. 2018;113:e180278. PubMed https://doi.org/10.1590/0074-02760180278

2. Cunha MS, da Costa AC, de Azevedo Fernandes NCC, Guerra JM, Dos Santos FCP, Nogueira JS, et al. Epizootics due to yellow fever virus in São Paulo State, Brazil: viral dissemination to new areas (2016–2017). Sci Rep. 2019;9:5474. PubMed https://doi.org/10.1038/s41598-019-41950-3

3. Cunha MDP, Duarte-Neto AN, Pour SZ, Pereira BBS, Ho YL, Perondi B, et al. Phylogeographic patterns of the yellow fever virus around the metropolitan region of São Paulo, Brazil, 2016–2019. PLoS Negl Trop Dis. 2022;16:e0010705. PubMed https://doi.org/10.1371/journal.pntd.0010705

4. Secretaria da Saúde —Governo do Estado de São Paulo. Dados Estatísticos [cited 2025 Apr 7]. http://saude.sp.gov.br/cve-centro-de-vigilancia-epidemiologica-prof.-alexandre-vranjac/areas-de-vigilancia/doencas-de-transmissao-por-vetores-e-zoonoses/agravos/febre-amarela/dados-estatisticos

5. Ho YL, Joelsons D, Leite GFC, Malbouisson LMS, Song ATW, Perondi B, et al.; Hospital das Clínicas Yellow Fever Assistance Group. Severe yellow fever in Brazil: clinical characteristics and management. J Travel Med. 2019;26:taz040. PubMed https://doi.org/10.1093/jtm/taz040

6.Escolani L, Florence B, Denaro M, Alexandre M. Isolation and complete sequence of a functional human glyceraldehyde-3-phosphate dehydrogenase gene. J Biol Chem. 1988; 263: 15335–15341.

7. White TJ, Brun T, Lee S, Taylor J. Amplification and direct sequencing of fungal ribosomal RNA genes for phylogenetics. In: Innis MA, Gelfand DH, Sninsky JJ, White TJ, editors. PCR protocols. A guide to methods and applications. San Diego: Academic Press; 1990. p. 315–20.

8. Fujita SI, Senda Y, Nakaguchi S, Hashimoto T. Multiplex PCR using internal transcribed spacer 1 and 2 regions for rapid detection and identification of yeast strains. J Clin Microbiol. 2001; 39: 3617–22. https://doi.org/10.1128/JCM.39.10.3617-3622.2001 PMID: 11574582.

9. Nadkarni MA, Martin FE, Jacques NA, Hunter N. Determination of bacterial load by real-time PCR using a broad-range (universal) probe and primers set. Microbiology (Reading). 2002;148:257–66. PubMed https://doi.org/10.1099/00221287-148-1-257

10. Deutch S, Dahlberg D, Hedegaard J, Schmidt MB, Møller JK, Ostergaard L. Diagnosis of ventricular drainage-related bacterial meningitis by broad-range real-time polymerase chain reaction. Neurosurgery. 2007;61:306–12. PubMed https://doi.org/10.1227/01.NEU.0000255526.34956.E4

Appendix Table. Molecular and histological data for microorganisms found in the lungs collected at autopsy of patients with severe yellow fever, São Paulo, Brazil 2017–2019*

N	Organism bacteria	%	GenBank accession no.	Gram	Organism fungi	%	GenBank accession no.	Histology					Concordance molecular test with histology
								Coccus	Gnbacilli	Yeast	Hyphae	BCP	
1	Negative				Negative			0	0	0	0	0	NA
2	Negative				Negative			0	0	0	0	1	NA
3	Negative				Negative			0	0	0	1	1	NA
4	Negative				Negative			0	0	0	0	0	NA
5	Negative				Negative			1	1	0	0	1	NA
6	Negative				Negative			0	1	1	0	1	NA
7	*Pseudomonas aeruginosa*	100	MN429315	Negative	*Nakaseomyces glabratus#*	99.7	MN473880	1	1	1	0	1	YES
8	*Acinetobacter baumannii*	98	MN428414		Negative			0	1	0	0	1	YES
9	*Streptococcus pneumoniae*	100	MN431202	Positive	*Debaryomyces hansenii*	98	MN472906	0	0	1	0	1	YES†
10	Negative				*Didymella glomerata*	98.7	MN472927	0	1	1	0	1	YES
11	*Serratia marcescens*	97	MN431240	Negative	Negative			0	1	0	0	1	YES
12	*Cronobacter dublinensis*	97	MN431459	Negative	Negative			0	1	0	0	1	YES
13	*Acinetobacter baumannii*	100	MN435153	Negative	Negative			0	1	0	0	1	YES
14	Negative				*Candida albicans*	100	MN473077	1	1	1	0	1	YES
15	*Enhydrobacter aerosaccus*	99	MN436791	Negative	Negative			0	1	0	0	1	YES
16	Negative				Negative			0	1	0	0	1	NA
17	*Klebsiella pneumoniae*	100	MN437654	Negative	Negative			0	1	0	0	1	YES
18	*Escherichia coli*	100	MN436844	Negative	Negative			0	1	1	0	1	YES
19	Negative				Negative			0	1	0	1	1	NA
20	*Moraxella osloensis*	99.76	MN437656	Negative	Negative			0	1	1	0	1	YES
21	*Escherichia coli*	97	MN437322	Negative	Negative			0	1	0	0	1	YES
22	*Enhydrobacter aerosaccus*	97.91	MN439924	Negative	Negative			0	1	1	0	1	YES
23	Negative				Negative			0	1	1	0	1	NA
24	*Enhydrobacter aerosaccus*	100	MN44786	Negative	*Aspergillus fumigatus*	96.9	MN474008	0	1	0	1	1	YES
25	Negative				Negative			0	0	1	0	0	NA
26	*Klebsiella pneumoniae*	99	MN44823	Negative	Negative			0	1	0	0	1	YES
27	Negative				Negative			0	1	0	0	0	NA
28	*Simiduia agarivorans*	97	MN447394	Negative	Negative			0	1	0	0	1	YES
29	Negative				Negative			0	1	0	0	1	NA
30	Negative				*Nakaseomyces glabratus*	100	MN475751	0	1	1	1	1	YES
31	*Acinetobacter* spp.	97	MN445371	Negative	Negative			0	1	0	0	1	YES
32	Negative				Negative			0	1	0	0	1	NA
33	Negative				Negative			0	0	1	0	1	NA
34	Negative				Negative			0	1	0	0	1	NA
35	Negative				Negative			0	1	0	0	1	NA

N	Organism bacteria	%	GenBank accession no.	Gram	Organism fungi	%	GenBank accession no.	Histology					Concordance molecular test with histology
								Coccus	Gnbacilli	Yeast	Hyphae	BCP	
36	*Pseudomonas aeruginosa*	99.49	MN447128	Negative	*Aspergillus fumigatus*	100	MN475197	0	1	0	1	1	YES
37	*Klebsiella pneumoniae*	99.75	MN447127	Negative	Negative			0	1	0	0	1	YES
38	Negative				Negative			0	0	1	0	0	NA
39	*Mycoplasma salivarium*	97.54	MN447214		*Candida albicans*	100	MN475275	0	1	1	0	1	YES
40	*Klebsiella pneumoniae*	99.58	MN526933	Negative	Negative			0	1	0	0	1	YES
41	*Enhydrobacter aerosaccus*	99	MN447222	Negative	Negative			0	1	0	0	1	YES
42	*Mycoplasma orale*	100	MN447304		Negative			0	1	0	0	1	YES
43	*Enhydrobacter aerosaccus*	100	MN447318	Negative	Negative			0	1	0	0	1	YES
44	*Klebsiella pneumoniae*	99	MN447416	Negative	*A. Fumigatus*	100	MN477796	0	1	0	0	1	YES‡
45	Negative				*Cladosporium sphaerospermum*	100	MN476933	0	0	0	0	1	NO
46	*Klebsiella pneumoniae*	99	MN447588	Negative	*Apiotrichum domesticum*	98.8	MN477197	1	1	0	0	1	YES§
47	Negative				*Aspergillus flavus*	100	MN477210	0	0	0	1	1	YES
48	Negative				Negative			0	0	0	1	1	NA
49	*Klebsiella pneumoniae*	100	MN447652	Negative	*Nakaseomyces glabratus*	99.5	MN477933	0	1	1	0	1	YES
50	*Klebsiella pneumoniae*	100	MN447666	Negative	Negative			0	1	0	0	1	YES
51	*Enhydrobacter aerosaccus*	100	MN447732	Negative	Negative			0	1	0	0	1	YES
52	*Pseudomonas baetica*	97	MN447668	Negative	Negative			0	1	0	0	1	YES
53	Negative				Negative			0	1	0	0	1	NA
54	Negative				*Candida tropicalis*	99.6	MN477465	0	1	1	0	1	YES
55	*Enhydrobacter aerosaccus*		MN441757	Negative	Negative			0	1	0	1	1	YES
56	Negative				Negative			0	0	0	0	1	NA
57	Negative				Negative			0	0	0	1	1	NA
58	Negative				Negative			0	1	0	0	0	NA
59	Negative				*Didymella glomerata*	99.3	MN472744	0	0	1	0	0	YES¶
60	*Enterobacter asburiae*	99	MN430860	Negative	*Trichosporon faecale*		MN472741	0	1	1	0	1	YES
61	*Acinetobacter baumannii*	99	MN431185	Negative	Negative			0	1	0	0	1	YES
62	*Enhydrobacter aerosaccus*	98	MN431431	Negative	Negative			0	1	0	0	0	YES
63	Negative				Negative			0	1	1	0	1	NA
64	*Klebsiella pneumoniae*	99	MN442076	Negative	Negative			0	1	0	0	1	YES
65	*Enhydrobacter aerosaccus*	99	MN44855	Negative	Negative			0	1	0	0	0	YES
66	*Moraxella osloensis*	96	MN445979	Negative	*Trichosporon asahii*	98.3	MN475174	1	1	1	0	1	YES
67	*Enhydrobacter aerosaccus*	100	MN445607	Negative	*Candida tropicalis*	100	MN475173	0	1	1	0	0	YES
68	Negative				Negative			0	1	1	0	0	NA

N	Organism bacteria	%	GenBank accession no.	Gram	Organism fungi	%	GenBank accession no.	Histology					Concordance molecular test with histology
								Coccus	Gnbacilli	Yeast	Hyphae	BCP	
69	*Enhydrobacter aerosaccus*	99	MN447234	Negative	*Nakaseomyces glabratus*	99.7	MN475749	1	1	1	0	1	YES
70	*Enhydrobacter aerosaccus*	97	MN447305	Negative	Negative			0	1	1	0	1	YES
71	*Enhydrobacter aerosaccus*	99	MN447407	Negative	*Candida albicans*	97	MN477032	0	1	1	0	1	YES
72	*Acinetobacter baumannii*	100	MN447636	Negative	*Candida parapsilosis*	99.3	MN477247	0	1	1	0	1	YES
73	Negative				Negative			0	1	1	0	1	NA

*BCP, bronchopneumonia; Gnbacilli, Gram-negative bacilli; N, case number; NA, not applicable (molecular test did not amplified DNA for bacteria or fungi)
†In this case, the histopathology did not detect gram-positive coccus, corresponding to *S. pneumoniae*, as detected by PCR.
‡In this case, the histopathology did not detect hyphae corresponding to *A. fumigatus* as detected by PCR.
§In this case, the histopathology did not detect hyphae corresponding to *A. domesticum* as detected by PCR.
¶ In this case, yeasts were identified in the pulmonary tissue but not associated with pneumonia, corresponding to terminal bronchoaspiration of microorganisms.
#Formerly *Candida glabrata.*
